# Barriers and proposed solutions to at‐home colorectal cancer screening tests in medically underserved health centers across three US regions to inform a randomized trial

**DOI:** 10.1002/cam4.70040

**Published:** 2024-08-08

**Authors:** Suzanne Brodney, Roopa S. Bhat, Jessica J. Tuan, Gina Johnson, Folasade P May, Beth A. Glenn, Kimberly Schoolcraft, Erica T. Warner, Jennifer S. Haas

**Affiliations:** ^1^ Division of General Internal Medicine, Massachusetts General Hospital Boston Massachusetts USA; ^2^ UCLA Kaiser Permanent Center for Health Equity, UCLA Center for Cancer Prevention and Control Research Jonsson Comprehensive Cancer Center Los Angeles California USA; ^3^ Community Health Prevention Programs Great Plains Tribal Leaders' Health Board Rapid City South Dakota USA; ^4^ Department of Medicine, David Geffen School of Medicine, UCLA Ronald Reagan Medical Center University of California Los Angeles Los Angeles California USA; ^5^ Vatche and Tamar Manoukian Division of Digestive Diseases, Department of Medicine, David Geffen School of Medicine University of California Los Angeles Los Angeles California USA; ^6^ Greater Los Angeles Veterans Affairs Healthcare System Los Angeles California USA; ^7^ Department of Health Policy and Management UCLA Fielding School of Public Health Los Angeles California USA; ^8^ Fight Colorectal Cancer Springfield Missouri USA; ^9^ Clinical and Translational Epidemiology Unit Massachusetts General Hospital and Harvard Medical School Boston Massachusetts USA; ^10^ Mongan Institute, Massachusetts General Hospital and Harvard Medical School Boston Massachusetts USA

**Keywords:** colorectal cancer screening, community health centers, disparities, FIT, FIT‐DNA, qualitative, tribal health facility

## Abstract

**Introduction:**

At‐home colorectal cancer (CRC) screening is an effective way to reduce CRC mortality, but screening rates in medically underserved groups are low. To plan the implementation of a pragmatic randomized trial comparing two population‐based outreach approaches, we conducted qualitative research on current processes and barriers to at‐home CRC screening in 10 community health centers (CHCs) that serve medically underserved groups, four each in Massachusetts and California, and two tribal facilities in South Dakota.

**Methods:**

We conducted 53 semi‐structured interviews with clinical and administrative staff at the participating CHCs. Participants were asked about CRC screening processes, categorized into eight domains: patient identification, outreach, risk assessment, fecal immunochemical test (FIT) workflows, FIT‐DNA (i.e., Cologuard) workflows, referral for a follow‐up colonoscopy, patient navigation, and educational materials. Transcripts were analyzed using a Rapid Qualitative Analysis approach. A matrix was used to organize and summarize the data into four sub‐themes: current process, barriers, facilitators, and solutions to adapt materials for the intervention.

**Results:**

Each site's process for stool‐based CRC screening varied slightly. Interviewees identified the importance of offering educational materials in English and Spanish, using text messages to remind patients to return kits, adapting materials to address health literacy needs so patients can access instructions in writing, pictures, or video, creating mailed workflows integrated with a tracking system, and offering patient navigation to colonoscopy for patients with an abnormal result.

**Conclusion:**

Proposed solutions across the three regions will inform a multilevel intervention in a pragmatic trial to increase CRC screening uptake in CHCs.

## Graphical abstract

Fifty‐three qualitative interviews were conducted with participants across 10 community health centers that serve medically underserved groups in three US regions to characterize current screening processes from identification of eligible patients through follow‐up of abnormal screening findings. Data were used to plan the implementation of a pragmatic randomized trial to compare mailed fecal immunochemical testing (FIT) versus FIT‐DNA.

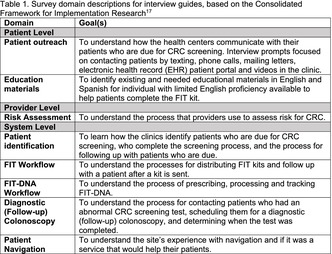

## 1 INTRODUCTION

Screening for colorectal cancer (CRC) can lower incidence and CRC mortality, but approximately one‐third of US adults are overdue for screening.^1^, ^2^ People from historically minoritized racial and ethnic populations have lower uptake of CRC screening, higher incidence and higher mortality.^3^, ^4^ Community Health Centers (CHCs) serve 1 in 12 people in the United States, and are critical to addressing health disparities.^5^, ^6^ Although CHCs are dedicated to providing evidence‐based care, they are often under‐resourced. Medically underserved populations, like those served in CHCs, have among the lowest CRC screening rates.^7^ In CHCs stool‐based screening is a common strategy.^8^ Two recent events have complicated delivery of CRC screening. First, the COVID‐19 pandemic resulted in large numbers of individuals overdue for screening.^9^ Second, millions of younger Americans are newly eligible for screening after the US Preventive Services Task Force lowered the recommended age to start CRC screening.^10^

Fecal immunochemical test (FIT) and FIT‐DNA are the most commonly used stool‐based screening modalities. While there are many FDA approved FIT brands, currently, Cologuard (Exact Sciences, Madison, WI) is the only FDA‐approved FIT‐DNA option. Stool‐based tests can be sent to a patient's home and returned by mail. FIT can also be given to a patient at a visit with a return mailer.^11^ FIT needs to be completed annually and a FIT‐DNA every 3 years. FIT can be used as a population‐based approach and recommendations exist to help implement this approach in clinical practice, including CHCs.^12^, ^13^, ^14^ However, little has been published on population‐based approaches to implementing FIT‐DNA.^15^ For both stool‐based screening options, an abnormal result must receive a follow‐up colonoscopy.

The goal of this study was to identify provider‐ and system‐level barriers and solutions to FIT and FIT‐DNA CRC screening in CHCs from the perspective of CHC clinical and administrative staff using qualitative methods. Findings would inform a pragmatic randomized trial to assess the effectiveness of mailed FIT or FIT‐DNA outreach in participating CHCs in three US geographic regions, and help to understand site‐based differences, create local adaptations to implementation strategies, and develop patient materials.

## 2 MATERIALS AND METHODS

Fifty‐three qualitative interviews were conducted with clinical and administrative staff from 10 CHCs in Boston, Massachusetts, Los Angeles, California, and South Dakota from March–September 2022. This project was conducted as the initial phase of a larger study. Investigators from clinical settings that serve low income and medically underserved patients in each region decided to collaborate for this work. These three regions differ tremendously with regards to geography, population density and population characteristics, which enhances the generalizability of findings beyond work in any one setting. The four Massachusetts CHCs are located in socioeconomically disadvantaged urban neighborhoods and are part of the primary care network of a large, academic integrated delivery system. In California, the four Federally Qualified Health Centers (FQHCs) are part of a single, centrally administrated FQHC system. In South Dakota, one tribally owned clinic (urban setting) and one Indian Health Service facility (rural setting) participated. At the South Dakota sites, patients are referred to as relatives because, in the Lakota culture, kinship is valued, and people are all connected to one another and understand that all life is sacred. Throughout, the term relative is used when referring to participants from South Dakota.

A purposive sampling approach was used by contacting medical and administrative directors at each site to identify staff in key roles who were involved with the CRC screening process. Research staff sent up to three emails to potential participants to inform them about the study. If the subject responded, an interview was scheduled. Snowball sampling was used to identify additional participants. The number of interviews conducted per site was based on representation of different roles and inclusion from key stakeholders in the CRC screening workflow until saturation in data collection was reached indicating redundancy in the information being reported at each site for each domain.^16^ Study procedures were approved by each site's Institutional Review Board.

The Consolidated Framework for Implementation Research^17^ was used to guide the development of the interview guides by focusing on patient, provider and system domains relevant to trial implementation (Table 1). At the patient level, we focused on how CHCs communicate with patients about CRC screening (mail, text, phone, electronic health record (EHR) patient portal), and what, how, and when educational materials are shared. FIT‐DNA screening is currently delivered by the company that makes the test using a standardized outreach protocol that includes follow‐up texts and phone calls to remind patients to complete and return the kit.^18^ We sought to understand if there were standardized outreach protocols at each site and if so, what components they included. At the provider level, we inquired about how providers identify CRC risk. At the system level, we explored processes to identify and track patients due or overdue (hereafter referred to as “due”) for CRC screening. We also asked about the process to prescribe, process and track FIT and FIT‐DNA kits, the workflow for patients who need a follow‐up colonoscopy after an abnormal stool test and the site's experience with patient navigation.

**TABLE 1:** Survey domain descriptions for interview guides, based on the Consolidated Framework for Implementation Research.^17^

Domain	Goal(s)
Patient level	
Patient outreach	To understand how the health centers communicate with their patients who are due for CRC screening. Interview prompts focused on contacting patients by texting, phone calls, mailing letters, electronic health record (EHR) patient portal and videos in the clinic
Education materials	To identify existing and needed educational materials in English and Spanish for individual with limited English proficiency available to help patients complete the FIT kit
Provider level	
Risk assessment	To understand the process that providers use to assess risk for CRC
System level	
Patient identification	To learn how the clinics identify patients who are due for CRC screening, who complete the screening process, and the process for following up with patients who are due
FIT workflow	To understand the processes for distributing FIT kits and follow up with a patient after a kit is sent
FIT‐DNA workflow	To understand the process of prescribing, processing and tracking FIT‐DNA
Diagnostic (follow‐up) colonoscopy	To understand the process for contacting patients who had an abnormal CRC screening test, scheduling them for a diagnostic (follow‐up) colonoscopy, and determining when the test was completed
Patient navigation	To understand the site's experience with navigation and if it was a service that would help their patients

### 2.1 Data collection

The interview guide was created and used across all sites (Supplement 1—Data S1). The summary template (Supplement 2—Data S1) and site matrix (Supplement 3—Data S1) were developed and tested in Massachusetts and then shared with the other sites. To test the interview guide, three pilot interviews were conducted in Massachusetts that included a physician, a project manager and a nurse in clinics not included in the interviews. All interviews in Massachusetts were conducted via a secure Teams account by SB and RS, and interviews in California were conducted virtually via a secure Zoom account by JT. Interviews typically lasted 30–45 minutes. Participants gave verbal consent to record interviews. The transcription feature embedded in the video tool was used to transcribe interviews, which were then edited based on notes taken during the interview, with re‐listening to recordings, as needed. Participants were sent a $50 gift card as an acknowledgement of their time and effort. The interviews in South Dakota were conducted by GJ and JA in‐person and virtually using Zoom. Participants were not paid for their time due to federal guidelines that prohibit employees from accepting payments.

### 2.2 Data analysis

The Rapid Qualitative Analysis Technique Assessment Process^19^ was used for analysis. This qualitative approach was selected because data from each site was necessary to inform the trial design and this allowed us to quickly summarize the results across topics and regions. One staff member (SB) with prior experience in qualitative analysis was trained on the Rapid Analysis method and conducted virtual training sessions via Teams with staff in California and South Dakota. The interviewers from each site met regularly via Teams to discuss the data.

After all the interviews were collected at each site, a two‐page summary was created for each interview from the transcript using the previously tested summary template (Supplement 2—Data S1: Example Summary Template). Five interviews from one of the Massachusetts CHCs were double coded by the two interviewers. Discrepancies were resolved through discussion and reviewing the recorded transcript. Since agreement was high for the initial five interviews that were double coded, the remaining Massachusetts interviews were coded by only one interviewer. Next, the main findings were organized into a site matrix for Massachusetts (Supplement 3—Data S1: Site Matrix). The process was carried out similarly in California by one interviewer who coded the interviews and had the Massachusetts interviewers review to ensure reliability. Discussions took place between the three interviewers about the similarities and differences in the CHC workflows between the two geographic regions. In South Dakota, the interviewers discussed coding results to identify inter‐site similarities and differences. The Standards for Reporting in Qualitative Research (SRQR) checklist was used to guide documentation of study components.^20^

## 3 RESULTS

### 3.1 Characteristics of the key informants

Across all sites, 53 interviews were conducted with clinic staff in a range of roles: 34% with nurses, 23% with primary care practitioners (PCPs), 15% with medical assistants, 28% with administrative staff and “other” (laboratory or quality improvement staff).

Feedback for each domain has been summarized and translated into solutions for how to adapt the methods and materials for the trial (Table 2).

**TABLE 2:** Considerations and solutions to adapt the methods and materials for trial implementation based on interview data.

Trial implementation	Exemplary quotes	Considerations	Solution for trial implementation
*Patient level*			
Patient outreach	“There's a lot of room for improvement in terms of tracking and following up and having that warm touch with somebody speaking with them in their own language to just ensure the visit has been completed or the visit is scheduled with reminders.” (Massachusetts [MA]) “If the patient returns for a clinic visit, then they are re‐educated and reissued a FIT test. If the patient needs outreach or does not return to us, and it's still non‐compliant, we will try to reach out to that patient again over the next couple of months, just going back through our outreach list and trying to reissue that patient and provide more education if we do. Not necessarily a standard set of reminders just because there's so many patients and so little resources to handle that.” (California [CA]) “I heard loud and clear that gas cards, you know, making them an incentive is really important.” (South Dakota [SD])	Though phone calls were cited as the best method to reach patients, this approach was not feasible due to limited staffing resources in the clinics Patients are more responsive to outreach from their clinical care team or health center and a mailed letter was preferable to the patient portal In SD, one rural location spanned approximately 1970 square miles which made reaching people by mail and asking them to return the kit by mail not feasible because of the distance from the postal service Offering an incentive for completing the CRC screening was important in SD	Directly mail screening kits to patients/relatives with pre‐addressed, postage‐paid return Recruitment letter written at a 6th‐ to 8th‐grade reading level, printed on health center letterhead with the medical director signature Materials available in English and Spanish based on patient's preferred language Create 3 text messages (1 primer and 2 follow‐up) and a process to deliver and track them (MA and CA) Offer gift cards as an incentive for completion in SD site to improve completion rates At one SD site, there are concerns about mailing stool kits given lack of access to a post office, so an alternate intervention will be centered around kit distribution at community health events
FIT education	“I think [the biggest barrier is] us handing them a FIT test and expecting that they really get what they're supposed to be doing and why they're supposed to be doing it. And the importance of doing it.” (MA) “I have a plethora of resources outlining the need for follow‐up. But that's not related to this topic. It may be related to general chronic disease. But there is no there's nothing in regard to CRC.” (SD) “Patients don't understand the importance of the screening. We try to educate as much as possible, but you know most patients, aren't aware of why it's needed, what the risk is of not having it. So it's the overall patient education.” (CA)	There was a need for simpler written instructions, materials in languages other than English, or instructions with pictures or a video Some patients are embarrassed to complete the FIT kit or there is a stigma around stool that makes patients uncomfortable with completing the kit	Adapt a FIT kit instruction sheet that is primary picture‐based but includes easier‐to‐read English and Spanish instructions reviewed by patient partners on the study team Create a short video, customized by site, on how to complete the FIT kit that is posted on a public YouTube channel available in English and Spanish A QR code to the video will be added to the FIT instruction sheet 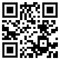 A link to the videos will be embedded in the text messages https://tinyurl.com/76r7yz74 In SD, a similar instructional video will be created to be shared through social media outlets
*Provider level*			
Risk assessment	“I think individual providers may use [risk assessment] tools. There's nothing that we do that's standardized across the practice…, I always am asking about family history. That's probably the number one risk assessment that I would do.” (MA) “It's basically just a history of present illness, family history, social history. So those kind of format what we usually do on annual physical. My practice is to usually ask everyone if they have any family cancer history, and if they say yes, I explore more, and then go from there.” (CA)	Across all the sites, providers used their clinical judgment to assess family history of cancer and prior history of polyps to determine if a patient was at average risk. There was no standardized tool used in practice	The MA and CA sites will collect data using the Prediction Model for gene Mutations (PREMM) algorithm, which is a clinical risk assessment tool. The PREMM will be administered by the patient navigator and takes less than 15 minutes to complete and will generate a score. The tool will be available in English and Spanish Create patient‐friendly handouts in English and Spanish that convert the PREMM score to a risk status that patients can share with their care team
*System level*			
Patient identification	“It would be nice if we had additional staffing to make those calls happen for people folks who are more overdue.” (MA) “Electronic health records do not interface. So if a patient was screened for CRC at another facility, you would not be notified through their chart.” (SD) “Honestly we don't actively do any patient panel screenings for colorectal cancer screening. It just comes up the day before [the appointment] that we have our MA sometimes help scrub the charts to take a look, but most of the time is just whoever shows up. We just try to make do our best to find who would be a fit for the screenings.” (CA)	There was no standardized report to identify patients who were due or overdue for CRC screening, but there were initiatives conducted at individual health centers	Develop a report that identifies eligible patients/relatives at each site to monitor their screening status and can be updated regularly to track the status of the kits from recruitment through colonoscopy, if needed Develop and share training materials across sites so staff and navigators are consistent in how they use the database
FIT workflow	“What would be super helpful… to find a process that just keeps circling back again and again to follow up on people and following up on results.” (MA) “Experience throughout the past ten years or so, [with the] FIT… Getting people to bring back their cards is always difficult.” (SD) “We don't really track whether they are returned or not. Once they're here, that's when we process them and that's when I get a message with the results to interpret or figure out the next steps for them.” (CA)	Across the sites, there was no standardized process in place to track who had received a FIT kit or the status of the kit Given staffing shortages it was not feasible for staff to send out the FIT kits, educate all the patients on how to complete and return the kit, or remind them that they had an outstanding kit	Develop a tracking system with standardized procedures that can be easily updated Purchase FIT kits directly from the vendor and study staff will send out via bulk mailing in MA and CA Include clear written instructions and the video in English and Spanish for how to complete the kit and send to the lab for processing Utilize text messages (1 primer and 2 follow‐up) to remind patients about the kit Perform a regular EHR query that will interface with the study database
FIT‐DNA workflow	“I'm starting to wonder if Cologuard is a better option because it's every three years as opposed to yearly. It's hard enough to get people to do it once, trying to get them to do it every year is challenging.” (MA) “I think that, there must be an educational piece that goes with it. So the folks understand what exactly they are, and what the whole process is.” (SD)	In MA, prior to the study, the ordering process for FIT‐DNA was a manual process that involved obtaining a provider's signature and faxing the order FIT‐DNA kits for this project were donated by the company so workflows had to be developed and implemented In CA and SD, prior to the study, FIT‐DNA was not widely offered at the health centers, so staff were not familiar with the product and were not aware it was an option	Use the company's portal to order and return the kits for this project Request data from the company to track the number of phone calls, text messages and emails in English and Spanish when kits are not promptly returned Import results from vendor's portal into the study database Implement workflows to integrate abnormal and negatives results to each site's EHR
Follow‐up colonoscopy	Patients get a printout for the prep with the instructions on it. “It tells you what to buy and what to do and when to do it, but … it's very wordy and you need to know what the stuff is that you're buying and you need to understand how to take it. And you need to understand how to do the diet ahead of time, the liquid diet. What does that mean? It's fairly complicated for someone who just has no or limited health literacy.” (MA) “We don't have a colonoscopy ability so we refer out to for a majority of the colonoscopies.” (SD) “We haven't had surgery [to perform colonoscopy] since I want to say more than five years…. Meanwhile, hospitals, are forty‐five minutes and fifty minutes away.” (SD) “I would say referral authorizations. That'd be the most difficult hurdle to get through coordination between [the site] and outside the facilities to have a colonoscopy performed, that's one of the biggest hurdles to get over as well.” (CA)	The workflows for how patients are contacted about the need for a follow‐up colonoscopy and the status of the appointment vary by site Patients often encounter barriers while scheduling the follow‐up colonoscopy, completing the bowel preparation, and arranging transportation	Rely on the routine workflow at each site to reach out to patients/relatives about the abnormal test Develop a process for patient navigators at each site to reach out to every patient/relative with an abnormal test to offer assistance with obtaining a follow‐up colonoscopy
Patient navigation	“This clinic would not run without our community health workers. So, if we feel that there's a concern around language barriers or just scheduling the appointment, we can easily refer them to see the CHW [community health worker]. Often times it's just one task. Follow through on the diagnostic colonoscopy. Ensure appointment is scheduled. Patient is reminded. Ensure that the appointment is completed. And if not, reschedule.” (MA) “Whenever they turn in their kits or whatever we are doing to make sure that we can capture their address and phone numbers so that when we must get a hold of them, if they have a positive iFOBT that we will be able to get a hold of them. We will have to go out and find people.” (SD) “I think if there were resources for a coordinator for sure, if there was an easy way for coordinators to access kits that weren't returned as an easy way to triage their time. I think it would be too much to call a patient up to remind them. Roughly 60–70% of the time patients will bring them back after at least 2 visits or so. Outside of those things, just having the extra support would be great.” (CA)	All clinics who had prior experience with patient navigators or community health workers were positive about the experience, but most of these positions had been funded by grants so were not long‐term positions The clinical and community resources available to help the navigator address patients' questions varied by site	Hire and train patient navigators at each site to contact each patients, in English or Spanish, with an abnormal FIT or FIT‐DNA result to help with scheduling or rescheduling a colonoscopy, taking the bowel preparation as instructed, and arranging transportation and answering any other questions the patients might have Navigators will complete the risk assessment with the patient and send them their risk score Track the status of patients who require outreach using the study database

### 3.2 Patient outreach (patient level)

Interviewers asked about methods of communicating with patients/relatives about the importance of CRC screening, being overdue for screening and how to do the test. Prompts were used to solicit feedback on specific methods including phone, texting, mailed letters, and patient portal. Phone calls were described as the preferred method of reaching patients/relatives in Massachusetts and California, but in South Dakota, in‐person visits were preferred. In Massachusetts and California, respondents expressed challenges reaching patients because verbal and written communication needed to include both English and Spanish, and some of the materials were not appropriate for low literacy populations. At all sites, there was enthusiasm for text messaging, though texting was only done for appointment reminders and the technology was not currently being used for screening reminders. Patients/relatives were reported to be more receptive to outreach from their specific clinic site compared to letters from the larger health system. Patient portals were uniformly perceived as ineffective for communication because many of the patients/relatives served either were not registered on the portal or were registered but were not active users. In South Dakota, most relatives received their FIT during in‐person visits, community health fairs events and appointments at the health care facility, and returned the kits to the clinic or through community health representatives. Health fairs, educational community events, and providing gift card incentives for gas or groceries were identified as preferred strategies for encouraging CRC screening.

### 3.3 FIT educational materials/instructions (patient level)

Interviewers asked respondents about the materials available to teach patients/relatives how to complete the FIT kit. A concern was that information included with the kits was difficult to understand and/or not available in languages other than English. In Massachusetts and California, sites described having clinical staff review instructions with a patient, but there were no standardized education tools or protocols. In these sites, clinical staff typically took out the kit, instructions, and mailer, and demonstrated how to complete the kit at home and instructed the patient to send it back.

### 3.4 Risk assessment processes (provider level)

FIT and FIT‐DNA are only indicated for people at average risk for CRC.^22^ Interviewers inquired about how risk is assessed and if any formal tools are used to confirm average risk status. Across sites, providers used their discretion when determining if a patient/relative was at average risk and suitable to complete FIT screening. None of the sites reported having any systematic protocols in place to assess risk assessment.

### 3.5 Patient identification (system level)

Respondents were asked about system‐level processes to identify patients/relatives due for CRC screening at each site. In all sites, providers reported checking the EHR to look for CRC screening care gaps at the time of a visit. At some of these sites, medical assistants reviewed the chart prior to an appointment and reminded the provider to order the CRC screening test (“scrubbing” the charts). At all sites, clinics performed EHR queries to determine who was due for screening to establish current screening rates, but these reports were not integrated into the workflow and a systematic process was not established to reach out to these patients.

### 3.6 FIT workflow (system level)

Interviewers inquired about how patients/relatives received a FIT kit and how staff documented when a FIT kit was returned. Though the process was highly variable, a provider typically ordered the kit at a visit, and the kit was either provided (in‐person visits) or mailed (virtual visits). In California, patients could pick up the kit and mail the completed kit back or hand‐deliver it to the lab. There were no standardized reminders to return the kit, but each site had a process in place to track the kit return. Following up on FIT kit orders and distribution requires staff time, and staffing shortages are universally a concern. Existing approaches included tracking expired orders in the EHR, providers setting individual reminders on their own calendar, using a physical notebook to log that a kit was sent so it was easier to determine who returned the kit, and changing the order expiration date, prompting a patient reminder call.

### 3.7 FIT‐DNA workflow (system level)

At the start of this study, FIT‐DNA was used in some Massachusetts health centers but was not available in the CHCs in California or South Dakota. In Massachusetts, FIT‐DNA was ordered via fax to Exact Sciences, but as this research began, these clinics initiated an EHR‐based ordering system. However, this project utilized donated kits so FIT‐DNA orders could not be placed through the EHR. FIT‐DNA kits were ordered using a company provided, HIPAA‐compliant portal, which required a new workflow to document results in the EHR and study database.

### 3.8 Follow‐up colonoscopy and patient navigation (system level)

Barriers exist to completing follow‐up colonoscopy, including difficulty with scheduling, time required, transportation, obtaining and following understandable instructions for the preparation, and fear of the colonoscopy findings. Tracking who is scheduled or has canceled their colonoscopy is difficult to determine without looking at individual charts. In Massachusetts and South Dakota, when an abnormal FIT or FIT‐DNA occurred, the result was sent to the provider and then the provider sent the patient/relative a letter or called to notify them of the result. The provider submitted an order for a colonoscopy and a nurse or medical assistant called the patient/relative to explain and schedule. In California, a patient with an abnormal result was called to schedule a follow‐up visit, during which the provider discussed the result. The clinic sent a letter with the results if the patient did not answer the phone after several attempts. Follow‐up colonoscopies were scheduled directly with gastroenterologists outside the CHC system.

At the South Dakota sites, providers must request an order to purchase “referred care” for a colonoscopy. This is a financial resource to assist with payment for healthcare services received by tribal citizens not available at a local tribal health facility. A service is either approved or disapproved based on available funds and population needs. Distances to the closest colonoscopy providers are over 100 miles away at one of the sites.

A patient navigator was identified as an essential support based on feedback about the follow‐up colonoscopy process. A navigator was not an existing position at the sites, but some sites had prior experience with navigators. There was a consistent sentiment that navigators are helpful and necessary, and sites universally reported that a patient navigator would facilitate follow‐up colonoscopy.

## 4 DISCUSSION

We conducted semi‐structured interviews with staff across three US regions to determine the patient‐, provider‐, and system‐level intervention components to implement in a pragmatic randomized trial to increase uptake of stool‐based CRC screening in CHCs. Across each domain, input on current processes and barriers were used to create a study protocol to be implemented across diverse settings and CHCs. Our findings address how to adapt and implement the methods and materials for a mailed, stool‐based, screening outreach intervention, especially in settings that lack resources and have staffing constraints. In addition to shaping our ongoing pragmatic trial, these components may suggest approaches that are reasonable in low resource settings.

Despite the findings about the preference for patients to receive outreach via telephone, we will contact patients via mail to inform them about the study, provide them with the opportunity to opt‐out, and provide CRC screening kits with supporting education materials in English and Spanish, including a video. The use of phone calls requires staff and resources and is not feasible. Use of a direct mail approach with postage‐paid envelopes for kit returns is supported by a systematic review that included these components in interventions to reach rural and low income populations.^25^ The use of kit completion reminders has also been identified as a highly effective strategy to increase CRC screening.^25^, ^26^ In the trial, we will include a text message primer message and two reminder text messages after the kit is sent. Since this is a pragmatic trial and there are staffing constraints in these settings, we believe this approach will be the least burdensome to the clinics and will provide an automated, sustainable alternative to manual tracking and phone outreach.

Results consistently identified a lack of educational information available in English and Spanish that is easy‐to‐understand. Limited health literacy has been identified as a barrier to completing CRC screening.^27^ Since the current system relies on healthcare workers to educate the patients about completing and returning the kit, developing materials that can be shared with patients, in a format they can access and understand, at the time it is needed, is crucial to minimize burden on the health system.^21^ The materials will be written at a 6th‐ to 8th‐grade reading level and we will utilize our patient partners, who are part of the study team, to review the materials. The videos, with subtitles, will be posted on a public site, which will help with comprehension.^28^

The trial incorporates a brief risk assessment using the Prediction Model for gene Mutations that will be administered to each patient with an abnormal screening test by a navigator.^22^, ^23^, ^24^ The PREMM model generates a score that is translated to average, medium or high risk of Lynch syndrome, or a status of not able to determine risk, if a patient is not familiar with the details of their family history. Since this is a pragmatic trial, personal or family history of colorectal polyps or CRC, as documented in the EHR, is being used to identify people ineligible for screening with a stool‐based test. However, an assessment of family history and genetic predisposition should be utilized to determine if stool‐based tests are appropriate for a particular patient. Currently, none of the sites use a standard risk assessment tool. If a person is considered high risk, genetic counseling, and testing are usually recommended practices, but patients from health centers are less likely to have complete family history information documented compared to tertiary care patients.^29^ Collecting data on feasibility of administering a risk assessment will inform future efforts in low resource settings.

We will address system level barriers by developing processes to identify and track patients, establish workflows for FIT and FIT‐DNA, and create a patient navigation system to help patients with an abnormal result receive a follow‐up colonoscopy. The database being developed for the study is user‐friendly, and training materials will include exemplar cases to ensure quality control and that each site uses the database consistently. The goal is to develop these protocols as part of a research study, but with attention to ensuring they are sustainable outside of a research setting. The results from each test will be integrated into each site's study database and EHR.

Established workflows at each site will be used to reach out to patients/relatives about the abnormal result. Patients/relatives across all sites with an abnormal screening result will be offered patient navigation to help each patient schedule a follow‐up colonoscopy. Navigation is an evidence‐based approach designed to ensure that necessary care is delivered to patients at risk for delays in care.^30^, ^31^ The resources for helping patients vary by site, so site‐specific resources will be created.

Limitations of this research include the inclusion of clinical and administrative staff who were identified by clinic leadership to participate, and did not include some roles within the health centers, such as front‐desk staff that may interact and answer patient questions about CRC screening and staff from the centers where patients/relatives obtain colonoscopy. Given the focus of the trial was to implement strategies at the provider and system level, we opted not to conduct interviews with patients. However, including patients likely would have enhanced the research. Even though the sites span three regions, the findings may not be generalizable. Despite these limitations, our qualitative study has many strengths. The three regions provide diverse perspectives of providers and staff who face similar and different challenges. We focused on an understudied and under‐resourced population. These findings provide insight into the barriers of CRC screening in CHCs to further mitigate health disparities in these communities.

## 5 CONCLUSIONS

This study gathered feedback from administrative and clinical staff across three US regions to help design a pragmatic randomized trial to increase participation in stool‐based CRC screening. Findings stress the importance of offering materials in English and Spanish, using text message reminders, adapting materials to address health literacy needs so patients can access instructions in writing, pictures or video, creating mailed workflows that integrate with each site's tracking system, and offering navigation to colonoscopy for all patients with an abnormal screening result.

## AUTHOR CONTRIBUTIONS

**Suzanne Brodney:** Conceptualization (equal); data curation (equal); formal analysis (equal); methodology (equal); project administration (equal); writing – original draft (equal); writing – review and editing (equal). **Roopa S. Bhat:** Conceptualization (equal); data curation (equal); formal analysis (equal); methodology (equal); project administration (equal). **Jessica J. Tuan:** Conceptualization (equal); data curation (equal); formal analysis (equal); project administration (equal); writing – original draft (equal). **Gina Johnson:** Data curation (equal); formal analysis (equal); project administration (equal); writing – original draft (equal). **Folasade P. May:** Data curation (equal); investigation (equal); methodology (equal); writing – review and editing (equal). **Beth A. Glenn:** Conceptualization (equal); methodology (equal); writing – review and editing (equal). **Kimberly Schoolcraft:** Conceptualization (equal); methodology (equal); writing – review and editing (equal). **Erica T. Warner:** Conceptualization (equal); methodology (equal); writing – review and editing (equal). **Jennifer S. Haas:** Conceptualization (equal); funding acquisition (equal); investigation (equal); methodology (equal); supervision (equal); writing – original draft (equal); writing – review and editing (equal).

## FUNDING INFORMATION

This work was supported by Stand Up To Cancer, a division of the Entertainment Industry Foundation and the American Cancer Society (CRP‐22‐080‐01‐CTPS).

## CONFLICT OF INTEREST STATEMENT

The authors disclose no conflicts of interest.

## ETHICS STATEMENT

The research was approved by the Mass General Brigham Institutional Review Board, the UCLA Institutional Review Board and the Great Plains Tribal Leaders’ Review Board.

## Supporting information


Data S1.

## Data Availability

Aggregate data that support the findings of this study are available from the corresponding author upon reasonable request with human subjects and data use approval.
