# Supplementary material for: Barriers and proposed solutions to at‐home colorectal cancer screening tests in medically underserved health centers across three US regions to inform a randomized trial
Source: Cancer Med. 2024 Aug 8;13(15):e70040. doi: 10.1002/cam4.70040 (PMC11310093; doi:10.1002/cam4.70040)
Supplement: Supplementary file 1 — Data S1. [file CAM4-13-e70040-s001.docx]

Supplement 1. Interview Guide

**Interview Guide – Clinical and Administrative Staff**

**Introduction:**

1. Will you please describe your role at the health center?
2. In what ways do you interact with CRC screening?
3. What CRC screening test options are available to patients at the health center?
   1. Which screening option do you think works better for patients?

**Eligibility/Identification:**

1. How does your clinic identify patients who are eligible for CRC testing?
2. Who does this process of identifying patients?
   1. Is there a population health manager or coordinator?
      1. Do you have pop health mgt software, if so, what system do you use?
3. How does your clinic identify patients who are overdue for CRC screening?
   1. Is there a report? If so, who runs the report and how often? Who gets the report?
4. Are patients contacted if they are overdue for screening?
   1. If so, how are they contacted?
   2. Are there members of your team who reach out to these individuals?

**FIT testing:**

1. What is the process for distributing FIT kits at your health center?

(Possible probe: Do you think patients prefer having kits mailed to them or have them be picked up at the clinic?)

1. Who delivers FIT education and what does it look like?
   1. Are there tools, videos, or handouts?
      1. Is there anything about these materials that you really like or that works well?
   2. Ask about use of videos in the clinic
2. How do you track FIT kit returns once they have been sent or given to a patient?
3. What is the process to follow-up with a patient if a kit is not returned?
   1. Are there standard reminders?
4. Do you receive kits that are returned as ‘undeliverable’?
   1. Does the clinic track this? Is there follow-up with the patient?
5. How often are the samples returned unusable?
   1. What happens when an unusable sample is returned?
   2. Does the clinic track this? Is there follow-up with the patient?

**Cologuard Testing:**

1. Does your health center use Cologuard?
2. What is the process for prescribing Cologuard?
3. What is the process of receiving the results from Cologuard?
4. How does the clinic keep track of whether a patient has completed the test?

**Risk Assessment:**

1. What is the process to assess whether a patient is at elevated risk for CRC?
   1. Prompts: Who does this, how is the history taken/recorded, how is this information used, what are the barriers?

**Diagnostic colonoscopy:**

1. For patients who have a positive test, what is the process for contacting them and scheduling a diagnostic colonoscopy?
2. How are patients informed of a positive result?
3. Where does the clinic refer patients to after a positive FIT or Cologuard? Is there a need to refer to different systems depending on patient insurance status?
4. How do you know if the patient has received the colonoscopy?
5. What, if anything, makes it difficult for patients who screen positive to get colonoscopies?
6. Who helps patients address barriers to getting a colonoscopy? (Community services? community health workers? Social workers? Patient navigators?)
7. Are there tools or resources that you use to educate patients about needed follow-up?
   1. If yes, is there anything about these resources that you think works really well?

**Contacting Patients:**

1. What concerns do you have about the Fit kit or Cologuard being mailed to the patients’ homes?
2. Does your clinic text patients? If so, for what? What is the response? What platform?
3. Do you have a sense of how comfortable patients are with using technology? For example, receiving texts, using QR codes, accessing video links.
4. When the health center contacts patients by mail, what format works best? (cover letter signed by physician, cover letter with health center’s address, phone calls, etc)

**General Practice:**

1. Is provider screening performance being tracked? Are there incentives?
2. Are there other studies and initiatives related to CRC screening going on in the clinics?

**Improvements:**

1. In your opinion, what do you think makes the process of CRC screening difficult for patients?
2. What, if anything, makes it easier for patients to get CRC screening?
3. Are there processes the clinic has tried in the past to increase CRC screening and follow up?
   1. What worked well? What didn’t?
4. How best can we best support you in improving the process of CRC screening in your clinic?
5. What do you think would be helpful to ensuring that patients who are overdue for follow-up of an abnormal screening test receive their follow-up?
6. Who else do we need to talk to from this clinic to get more information?
7. What would you like to share with us, that we didn’t ask about, that you think will be helpful to this project?

Supplement 2. Summary Template Form

STUDY ID #xx

Preparer/date:

Site:

Role:

Patient Outreach (phone, text, mail, Patient Gateway [PG])

Patient Identification

Risk Assessment

FIT workflow

FIT-DNA workflow

Diagnostic (Follow-up) colonoscopy

Patient navigation

Education materials

- *FIT materials*
- *Diagnostic (follow-up) colonoscopy materials*

General notes/takeaways:

Supplement 3. Sample Site Matrix Template

SITE

| Domain | Current process | Barriers/Challenges | Facilitators | Consensus |
| --- | --- | --- | --- | --- |
| Patient Outreach (phone, text, mail, Patient Gateway [PG]) |  |  |  |  |
| Patient Identification |  |  |  |  |
| Risk Assessment |  | N/A | N/A |  |
| FIT Workflow |  |  |  |  |
| FIT-DNA Workflow |  |  |  |  |
| Diagnostic (Follow-up) colonoscopy |  | **Patient barriers**  **Follow up**  **Scheduling**  **Travel**  **Prep** |  |  |
| Patient Navigation (PN) |  |  |  |  |
| Education materials | **FIT materials**  **Diagnostic (Follow-up) colonoscopy materials** |  |  | **FIT materials**  **Diagnostic (Follow-up) colonoscopy materials** |
